# Sleep Apnea Syndrome in an Elderly Population Admitted to a Geriatric Unit: Prevalence and Effect on Cognitive Function

**DOI:** 10.3389/fnagi.2019.00361

**Published:** 2020-01-10

**Authors:** Jadwiga Attier-Zmudka, Jean-Marie Sérot, Jeremy Valluy, Mo Saffarini, Youcef Douadi, Krzysztof Piotr Malinowski, Olivier Balédent

**Affiliations:** ^1^Department of Gerontology, Saint-Quentin Hospital, Saint-Quentin, France; ^2^CHIMERE, EA 7516 Head & Neck Research Group, University of Picardy Jules Verne, Amiens, France; ^3^ReSurg SA, Nyon, Switzerland; ^4^Department of Pulmonology, Saint-Quentin Hospital, Saint-Quentin, France; ^5^Institute of Public Health, Faculty of Health Sciences, Jagiellonian University Medical College, Kraków, Poland; ^6^BioFlowImage, Image Processing Unit, University Hospital of Amiens, Amiens, France

**Keywords:** sleep apnea, cognitive deficit, elderly population, OSAS, CSAS

## Abstract

**Background:**

Sleep apnea leads to cognitive impairment in older patients, but its association with neurodegeneration remains controversial, and most studies do not distinguish between the more common obstructive form (OSAS) and the rarer central form (CSAS).

**Objective:**

The purpose of this study was to assess the prevalence of the different forms of sleep apnea in a cohort of cognitively impaired elderly patients (>70 years) and to investigate their associations with cognitive deficit, weighted against known risk factors for neurodegeneration.

**Methods:**

Overnight polygraphy was performed for 76 consecutive patients admitted to our geriatric unit. Their cognitive function was assessed using the Mini Mental-State Exam (MMSE), Mattis Dementia Rating Scale (MDRS) and Stroop test. Multivariable analyses were performed to determine associations between cognitive function and independent variables describing demographics, sleep apnea measures, and cardiovascular risk factors.

**Results:**

The cohort comprised 58 women and 18 men aged a mean of 84 years (range, 73–96). Sleep apnea syndrome (SAS) was diagnosed in 48 patients (63%), of which 31 (41%) with OSAS and 17 (22%) with CSAS. Multivariable regression analysis revealed that MDRS was lower in patients with OSAS (β = −10.03, *p* = 0.018), that Stroop Colors and Words delays increased with AHI (β = 0.17, *p* = 0.030 and β = 0.31, *p* = 0.047) and that that Stroop Interference delay was higher in patients with CSAS (β = 24.45, *p* = 0.002).

**Conclusion:**

Sleep apnea is thus highly prevalent in elderly patients with cognitive impairment. OSAS was associated with lower general cognitive function, while CSAS was only associated with increased Stroop Interference delays. Elderly patients with cognitive deficit could benefit from sleep apnea screening and treatment.

## Introduction

Sleep apnea syndrome (SAS) is a disorder characterized by repeated pauses in breathing during sleep (Young et al., 1993). It is often underdiagnosed (Peppard et al., 2013), though its prevalence is increasing and was recently shown to be up to 40% in the general population (Heinzer et al., 2015), and likely higher in the elderly population (Al Lawati et al., 2009; Heinzer et al., 2015). SAS interrupts sleep and causes intermittent hypoxia (Rosenzweig et al., 2015) which could contribute to a range of pathophysiological consequences, including cognitive impairment (Wallace and Bucks, 2013; Arli et al., 2015), particularly in older patients (Yaffe et al., 2011; Osorio et al., 2015; Tsapanou et al., 2018). In fact, SAS is more prevalent in patients suffering from neurodegenerative diseases (Moran et al., 2005; Emamian et al., 2016) and is suspected to increase risks of developing Alzheimer’s disease (Aoki et al., 2014; Osorio et al., 2015; Liguori and Placidi, 2018).

The association between SAS and neurodegenerative diseases remains controversial (Somers et al., 2008; Rosenzweig et al., 2015) and the pathophysiological consequences of SAS are still unclear (Yaffe et al., 2011; Zimmerman and Aloia, 2012; Martin et al., 2015; Bubu et al., 2019). This is further complicated by the fact that most studies do not distinguish between the more common obstructive form (OSAS), characterized by recurrent collapse of the upper respiratory tract, and the rarer central form (CSAS), characterized by repetitive pauses in breathing without respiratory effort. Moreover, the causes of cognitive impairments are difficult to establish in patients over 70 years old, who often exhibit multiple co-morbidities.

Validated modifiable risk factors can be used to mitigate the progression of age-related cognitive decline. For instance, OSAS is associated with Alzheimer’s disease biomarker accumulation (Bubu et al., 2019) and can be treated with continuous positive airway pressure (CPAP) (Marshall et al., 2006; Cooke et al., 2009; Kylstra et al., 2013; Richards et al., 2019), which improves cognitive function in patients suffering from Alzheimer’s disease, although its effect on pathological progression remains unknown (Ancoli-Israel et al., 2008; Troussiere et al., 2014; Dostalova et al., 2019). The purpose of this exploratory study was therefore to (a) assess the prevalence of each form of SAS in a cohort of elderly patients (>70 years old) admitted to our geriatric unit for non-acute reasons, and (b) investigate the independent relation between sleep apnea and cognitive performance, measured using validated tests, including the Mini Mental-State Exam (MMSE), the Mattis Dementia rating Scale (MDRS) and the Stroop test, and competing against known risk factors for cognitive impairment. The hypothesis was that (i) the prevalence of SAS in elderly patients consulting for cognitive deficit would be greater than that reported for the general population and (ii) elderly patients with SAS would have significantly worse MMSE, MDRS and Stroop test scores, independently of confounding factors.

## Materials and Methods

**FIGURE 1:**
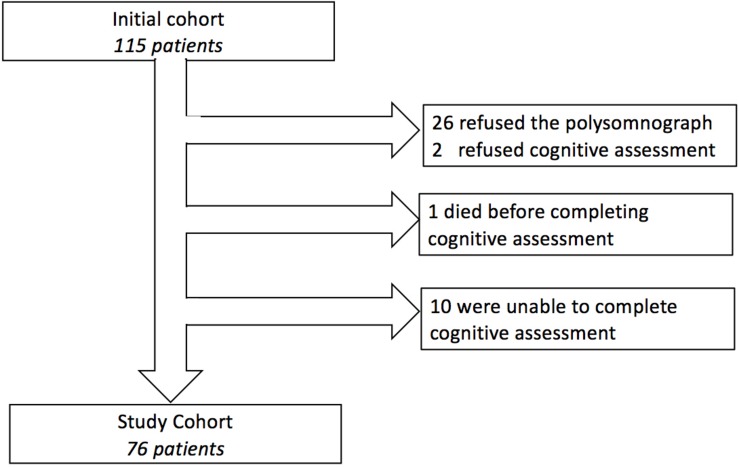
Flowchart of patient inclusions and exclusions.

We enrolled 115 consecutive patients admitted to our geriatric unit for non-acute reasons (non-emergency, no surgery, no mandatory overnight stay) between October 2015 and March 2018. The inclusion criterion was patients aged over 70 years. The exclusion criteria were (i) refusal to undergo sleep apnea diagnosis with overnight polygraph recording (26 patients), or (ii) refusal or inability to complete neurocognitive assessment using structural cerebral magnetic resonance imaging (MRI) (12 patients). In addition, 1 patient died before completing the cognitive assessment, which left a study cohort of 76 patients (Figure 1), who underwent overnight ambulatory polygraphy (Weinmann SOMNOlab 2, Hamburg, Germany) to measure their Apnea-Hypopnea Index (AHI), total number of events per night, mean arterial blood oxygen saturation (SaO_2_) and sleep time at SaO_2_ below 90% (Kuwabara et al., 2018). Apnea severity was noted based on the AHI, as mild (AHI 5–15), moderate (AHI 16–30), or severe (AHI > 30). Apneas were defined as cessation of airflow for at least 10 s and hypopneas were defined as a reduction of airflow to ≤50% of baseline for at least 10 s (Somers et al., 2008). The final diagnosis of sleep apnea, obstructive (OSAS) or central (CSAS) was determined by a pulmonologist (YD) based on the relative number of obstructive or central events per night, based on the definition by Somers et al. (2008): obstructive sleep apnea is characterized by ongoing ventilatory effort while central sleep apnea is characterized by withdrawal of central respiratory drive. OSAS and CSAS were defined when a patient had 5 or more episodes of obstructive or central apneas per hour of sleep, respectively.

The patient’s overall cognitive function was assessed by clinical psychologists (IG and ASM) using the following validated scores: (a) Mini-Mental State Examination (MMSE) (Kalafat Michel et al., 2003), which ranges from 0 to 30 points. Patients scoring ≤23 considered cognitively impaired. (b) MDRS, which comprises five subscales, spanning the areas of attention, initiation-perseveration, construction, conceptualization and memory (Hersch, 1979; Gardner et al., 1981). (Heinzer et al., 2015) Stroop Colors and Words test was used to assess attention and executive functioning (Comalli et al., 1962), which are known to be affected by sleep apnea (Olaithe and Bucks, 2013; Martin et al., 2015; Rosenzweig et al., 2015). The Colors test asks patients to recognize the color of dots that appear for a short time, while the Words test asks them to read words (names of colors) that appear for a short time. The Interference test requires the patients to read the words (names of colors) written in different (wrong) colors and repress the wrong answer (Stroop interference effect). For all tests, the delay before the answer, and the number of uncorrected mistakes, are reported. (Al Lawati et al., 2009) Montgomery-Asberg Depression rating scale [MADRS (Montgomery and Asberg, 1979)], on 60 points, and Goldberg anxiety scale (Huber et al., 1999) on nine points, to rule out effects of depression or anxiety on cognitive test results.

Final etiological diagnosis of cognitive impairment was based on the neurocognitive assessments, as well as MRI (Siemens Avanto 27780, Munich, Germany) results and history of geriatric consultations. Mild cognitive impairment was diagnosed following the criteria of Petersen et al. (2001), while Alzheimer’s disease (AD) and AD-like diseases were diagnosed according to the Diagnostic and Statistical Manual of Mental Disorders, 4th Edition (DSM-IV) and the recommendations of the National Institute on Aging – Alzheimer’s Association workgroups (McKhann et al., 2011).

Vascular risk factors for the development of AD (Yaffe et al., 2011) were diagnosed based on a comprehensive assessment performed in our geriatric unit. Blood samples were obtained after a minimum of 10 h of fasting. The diagnosis of vitamin D [25(OH)D_3_] deficiency was given if blood measurements were below 30 ng/mL, diabetes was diagnosed with blood glucose levels above 7 mmol/L and anemia with hemoglobin levels below 12 (women) or 13 (men) g/dL. Likewise, the reference range was 4.1–6.5 mmol/L for total cholesterol and 0.6–1.8 mmol/L for triglycerides. Inflammation was diagnosed if the blood levels of C-reactive protein (CRP) was higher than 10 mg/L (McMorris et al., 2017) as per the recommendations of the World Health Organization (World Health Organization [WHO], 2014), and leucocyte concentration was higher than 100000/mm^3^. The blood pressure of patients was measured during waking hours, and after polygraphy, during night-time hours.

Written informed consent was obtained from all patients for their participation and confirmed by their next-of-kin if necessary. The study protocol was approved by an independent Ethical Review Board (CPP Amiens: 2015/6) and the national Data Protection Authority (CNIL:150075B-31). The study was registered in clinicaltrials.gov (NCT02578303). All procedures were performed in accordance with the 1964 Helsinki declaration.

### Statistical Analysis

Descriptive statistics were used to summarize the data. Shapiro–Wilk tests were used to assess the normality of distributions. Due to skewed distribution of continuous variables, the data was presented as median [IQR]. Univariable regressions were performed to determine associations between five outcomes (MMSE, MDRS and Stroop Colors, Words, and Interference tests) and seventeen independent variables (age, BMI, sex, diagnosis of SAS, OSAS, or CSAS, AHI, sleep apnea severity, blood oxygen saturation, sleep time with hypoxia, number of events per night, diabetes, inflammation, hypercholesterolemia, hypertriglyceridemia, vitamin D deficiency, and anemia). Variables that do not measure sleep apnea were included because they represent cardiovascular risk factors known to affect sleep apnea or cognition in elderly patients (Fusetti et al., 2012). Multivariable regressions were then performed after backward selection (criterion *p* < 0.15). Hypercholesterolemia was excluded from the multivariable models due to a low incidence (*n* < 10) in our cohort. Sample size of 47 patients was calculated to be sufficient to show even the moderate negative correlation between the MDRS and the AHI with 80% power and 5% significance. Therefore, our sample size of 76 was deemed sufficient for analysis, considering a probably high failure rate in the neurocognitive tests due to the age of the patients. Statistical analyses were performed using R version 3.3.2 (R Foundation for Statistical Computing, Vienna, Austria). *P*-values < 0.05 were considered statistically significant.

## Results

The final cohort comprised 58 women (76%) and 18 men (24%), aged 84 [IQR, 80–88] years (range, 73–96), with BMI 24 [IQR, 21–27] kg/m^2^ (range, 15.6–40) (Table 1). Overall, they were not depressed (MADRS:6, IQR, 3–11; range, 0–31) nor anxious (Goldberg:3, IQR, 1–5; range, 0–9). A number of patients presented comorbidities, including heart failure (11.8%), aortic stenosis (3.9%), mitral stenosis (1.3%), carotid stenosis (3.9%), cerebral hematoma (3.9%), transient ischemic accident (3.9%), and cancer (25%). The prevalence of arterial hypertension was 32% while awake but rose to 59% while asleep. Thirty-three patients (43%) had mild cognitive impairment, 9 (12%) had vascular dementia, 7 (9%) had Alzheimer’s disease, 1 (1%) had Lewy-body dementia, and 26 (34%) had mixed dementia.

**TABLE 1:** Patient demographics.

	Cohort (*n* = 76)		
	Median	[IQR]	(Range)
Age (years)	84	[80–88]	(73–96)
BMI (kg/m^2^)	24	[21–27]	(16–40)
Depression (MADRS^∗^/60)	6	[3–11]	(0–31)
Anxiety (Goldberg/9)	3	[1–5]	(0–9)
	***n***	**(%)**	
Women	58	(76%)	
Diabetes	10	(13%)	
Hypercholesterolemia	5	(7%)	
Hypertriglyceridemia	18	(24%)	
Vitamin D deficiency	40	(53%)	
Anemia	51	(67%)	
Hypertension (awake)	23	(32%)	
Hypertension (asleep)	41	(59%)	
**Diagnosis**			
Mild cognitive impairment	33	(43%)	
Vascular dementia	9	(12%)	
Alzheimer’s disease	7	(9%)	
Dementia with Lewy bodies	1	(1%)	
Mixed dementia	26	(34%)	

*^∗^Montgomery-Asberg Depression Rating Scale. IQR, Interquartile range.*

The median AHI was 16.4 (IQR, 5.4–35.3; range, 0.5–73), and indicated severe sleep apnea in 32 patients (42%) (Table 2). SAS was diagnosed in 48 patients (63%), of which 31 (41%) with OSAS and 17 (22%) with CSAS.

**TABLE 2:** Sleep Apnea Syndrome.

	Cohort (*n* = 76)		
	Median	[IQR]	(Range)
Blood oxygen saturation (SpO_2_,%)	95	[93–96]	(87–98)
Sleep time with hypoxia^∗^(%)	2	[1–7]	(0–54)
Number of events per night	56	[22–113]	(2–548)
Apnea-hypopnea index (AHI)	16	[5–35]	(1–73)
	***n***	**(%)**	
**SAS severity (AHI**)			
None (<5)	19	(25%)	
Mild (5–15)	18	(24%)	
Moderate (15–30)	7	(9%)	
Severe (>30)	32	(42%)	
**Sleep Apnea Syndrome (SAS**)	48	(63%)	
Obstructive	31	(41%)	
Central	17	(22%)	

*^∗^Hypoxia defined as a reduction of SpO2 by 10%. IQR, Interquartile range.*

**FIGURE 2:**
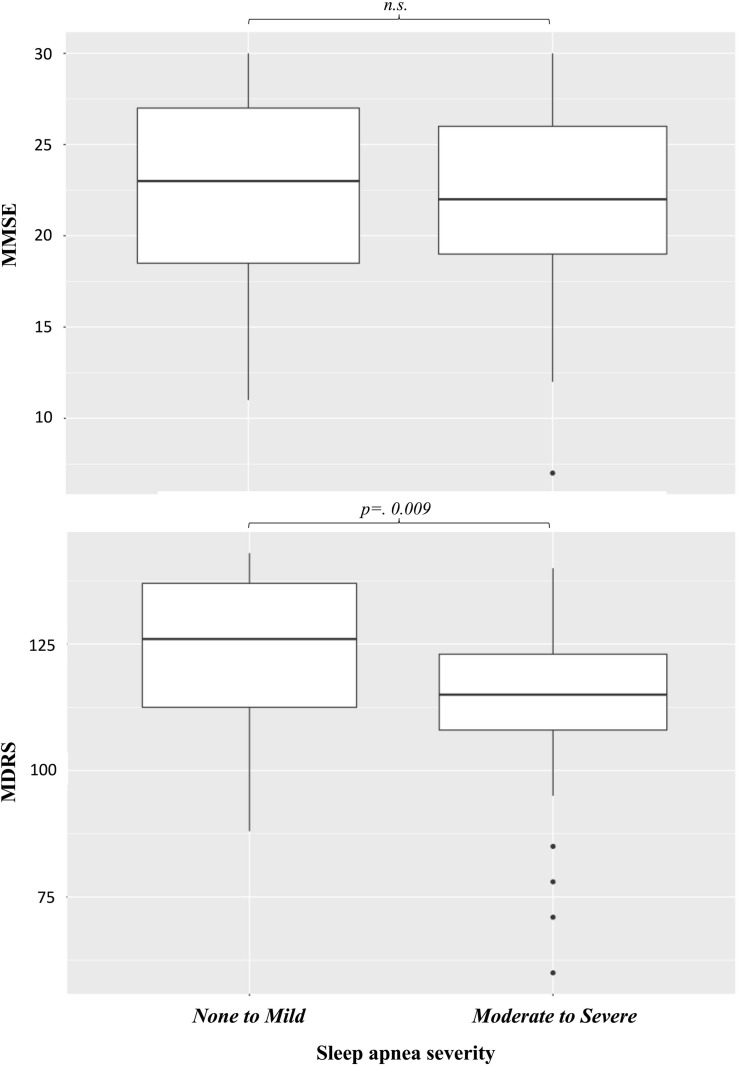
Effects of sleep apnea on cognitive scores.

The median MMSE score was 22.5 (IQR, 19–26; range, 7–30), with 41 (54%) patients scoring ≤23 (Table 3). Univariable regression revealed the MMSE score to be associated with sex and diabetes (Table 4). There was no significant difference in MMSE between patients with moderate to severe SAS and patients with mild to no SAS (Figure 2). Multivariable regression analysis revealed that MMSE decreased with age (β = −0.25, CI, −0.45 to −0.05; *p* = 0.014) and in patients with diabetes (β = −4.91, CI, −8.10 to −1.72; *p* = 0.01.

**TABLE 3:** Cognitive Tests.

	Cohort (*n* = 76)		
	Median	[IQR]	(Range)
Mini-mental state exam (MMSE/30)	23	[19–26]	(7–30)
Mattis dementia rating scale (MDRS/144)	121	[109–132]	(60–143)
Stroop color and word test			
Colors (delay, s)	19	[15–29]	(6–70)
Colors (non-corrected mistakes)	0	[0–0]	(0–6)
Words (delay, s)	34	[25–50]	(14–147)
Words (non-corrected mistakes)	0	[0–1]	(0–9)
Interference (delay, s)	53	[38–76]	(15–116)
Interference (non-corrected mistakes)	4	[1–13]	(0–24)

*IQR, Interquartile range.*

**TABLE 4:** Uni- and Multi-variable regression to identify factors associated with the Mini Mental State Exam (MMSE).

	Univariable			Multivariable (*n* = 71) Backward selection		
	Regression coefficient	95% C.I. (range)	*p*-Value	Regression coefficient	95% C.I. (range)	*p*-Value
Age	−0.19	(−0.39 to 0.02)	0.072	−0.25	(−0.45 to −0.05)	0.014
BMI (kg/m^2^)	0.14	(−0.09 to 0.37)	0.226			
Female Gender	−2.72	(−5.37 to 0.08)	0.044			
SAS	0.07	(−2.22 to 2.35)	0.955			
OSAS	−0.29	(−2.58 to 2.00)	0.801			
CSAS	0.92	(−1.86 to 3.70)	0.510			
Apnea-hypopnea index (AHI)	0.01	(−0.05 to 0.07)	0.723	0.04	(−0.01–0.10)	0.142
**Sleep apnea severity**						
*None*	REF					
*Mild*	1.11	(−2.08 to 4.31)	0.489			
*Moderate*	−3.44	(−7.90 to 1.01)	0.128			
*Severe*	0.35	(−2.46 to 3.15)	0.806			
Blood oxygen saturation (SpO2,%)	−0.07	(−0.60 to 0.45)	0.787			
Sleep time with hypoxia^∗^ (%)	0.01	(−0.07 to 0.10)	0.738			
Number of events per night	0.00	(−0.01 to 0.01)	0.438			
Diabetes	−4.02	(−7.30 to −0.73)	0.017	−4.91	(−8.10 to −1.72)	0.003
Inflammation	1.96	(−0.33 to 4.25)	0.092			
Hypercholesterolemia^∗∗^	−0.83	(−5.31 to 3.65)	0.712			
Hypertriglyceridemia	−0.89	(−3.58 to 1.79)	0.508			
Vitamin D deficiency	1.17	(−1.09 to 3.44)	0.304	1.83	(−0.33 to 4.00)	0.096
Anemia	0.17	(−2.26 to 2.59)	0.892			

*^∗^Hypoxia defined as a reduction of SpO2 by 10%. ^∗∗^Excluded from multivariable analysis due to low incidence (<10).*

The median MDRS score was 120.5 (IQR, 109.3–132.0; range, 60–143) (Table 3). Univariable regression revealed the MDRS to be associated with SAS, OSAS, AHI, diabetes, and hypertriglyceridemia (Table 5). The MDRS score was lower for patients with moderate to severe SAS compared to patients with mild to no SAS (Figure 2; *p* = 0.009). Multivariable regression analysis revealed that MDRS decreased with age (β = −0.81, CI, −1.54 to −0.08; *p* = 0.031), in patients with OSAS (β = −10.03, CI, −18.26 to −1.80; *p* = 0.018), and in patients with diabetes (β = −16.10, CI, −27.8 to −4.36; *p* = 0.008).

**TABLE 5:** Uni- and Multi-variable regression to identify factors associated with the Mattis Dementia Rating Scale (MDRS).

	Univariable			Multivariable (*n* = 66) Backward selection		
	Regression coefficient	95% C.I. (range)	*p*-Value	Regression coefficient	95% C.I. (range)	*p*-Value
Age	−0.74	(−1.52 to 0.04)	0.062	−0.81	(−1.54 to −0.08)	0.031
BMI (kg/m^2^)	0.14	(−0.77 to 1.06)	0.755			
Female Gender	−9.24	(−19.09 to 0.61)	0.066			
SAS	−8.78	(−17.50 to −0.05)	0.049			
OSAS	−9.94	(−18.72 to −1.17)	0.027	−10.03	(−18.26 to −1.80)	0.018
CSAS	2.74	(−7.35 to 12.84)	0.589			
Apnea-hypopnea index (AHI)	−0.23	(−0.44 to −0.01)	0.039			
**Sleep apnea severity**						
*None*	REF					
*Mild*	5.50	(−6.84 to 17.84)	0.377			
*Moderate*	−4.17	(−20.61 to 12.27)	0.614			
*Severe*	−9.36	(−20.06 to 1.33)	0.085			
Blood Oxygen Saturation (SpO2,%)	0.83	(−1.25 to 2.90)	0.430			
Sleep time with hypoxia^∗^ (%)	0.03	(−0.29 to 0.35)	0.865			
Number of events per Night	0.01	(−0.03 to 0.04)	0.745			
Diabetes	−13.42	(−25.87 to −0.97)	0.035	−16.10	(−27.8 to −4.36)	0.008
Inflammation	6.35	(−2.63 to 15.33)	0.163			
Hypercholesterolemia^∗∗^	−10.83	(−27.46 to 5.79)	0.198			
Hypertriglyceridemia	−9.99	(−19.91 to −0.07)	0.048			
Vitamin D deficiency	0.07	(−9.39 to 8.35)	0.907			
Anemia	−4.93	(−14.35 to 4.49)	0.300	−4.47	(−13.13 to 4.19)	0.306

*^∗^Hypoxia defined as a reduction of SpO2 by 10%. ^∗∗^Excluded from multivariable analysis due to insufficient number of observations (<10).*

**FIGURE 3:**
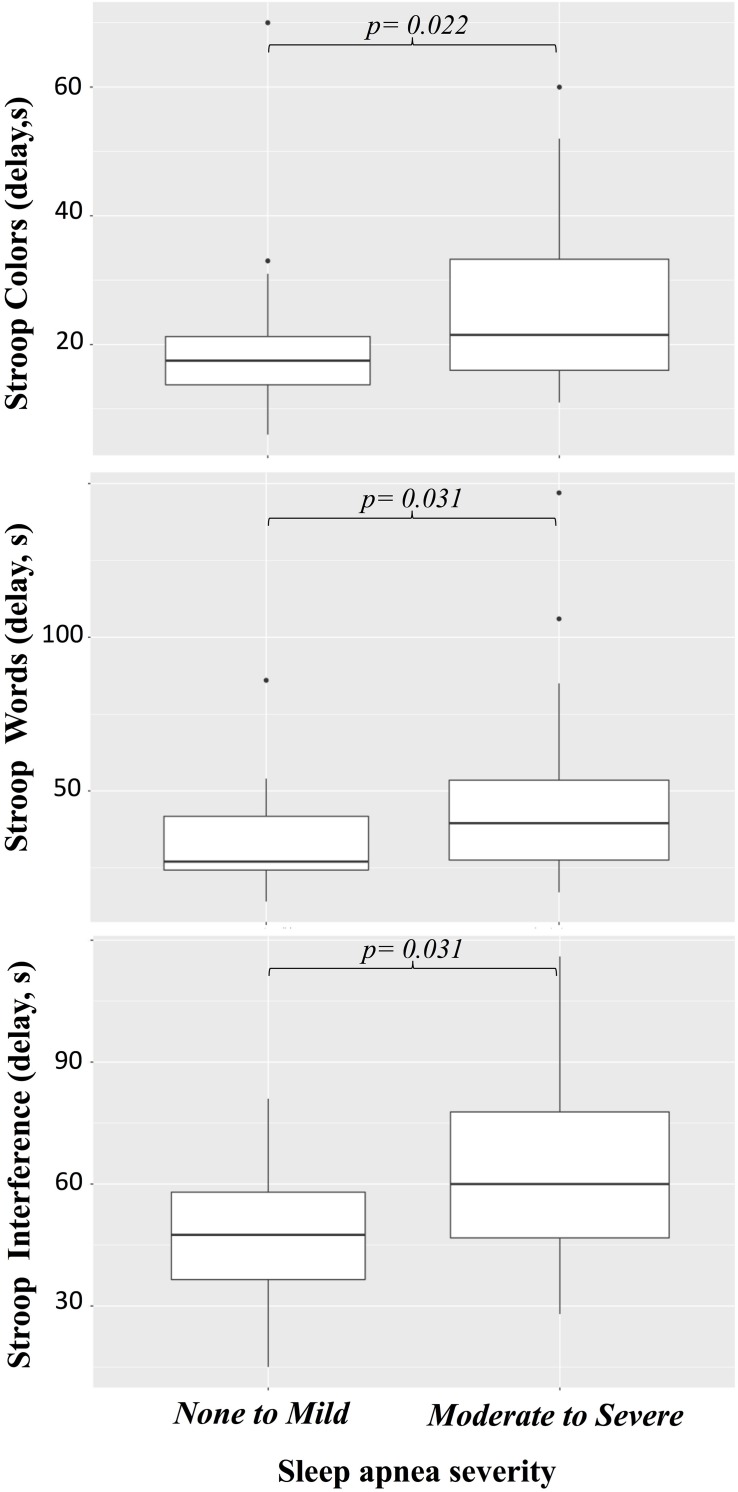
Effects of sleep apnea on Stroop tests.

Twenty patients were unable to perform the Stroop test. For the remaining patients, the median Stroop Colors delay was 19 s (IQR, 15–29; range, 6–70) and the median number of mistakes was 0 (IQR, 0–0; range, 0–6) (Table 3). The Stroop Words delay was 34 s (IQR, 25–50; range, 14–147) and the median number of mistakes was 0 (IQR, 0–1; range, 0–9). The Stroop Interference delay was 53 s (IQR, 38–76; range, 15–116) and the median number of mistakes was 4 (IQR, 1–13; range, 0–24). Univariable regressions revealed the Stroop Colors delay to be associated with age, AHI, severe SAS, and anemia (Table 6), the Stroop Words delay to be associated with AHI (Table 7), and the Stroop Interference delay to be associated with SAS, CSAS, AHI and diabetes (Table 8). All three delays were increased in patients with moderate to severe SAS compared to patients with mild to no SAS (Figure 3, Color, *p* = 0.022, Word, *p* = 0.031, Interference, *p* = 0.031). Multivariable regressions revealed that Stroop Color delay increased with age (β = 0.64, CI, 0.09–1.19; *p* = 0.024) and AHI (β = 0.17, CI, 0.02–0.32; *p* = 0.030), that Stroop Words delay increased with AHI (β = 0.31, CI, 0.00–0.62; *p* = 0.047) and that Stroop Interference delay increased in patients with CSAS (β = 24.45, CI, 15.21–50.55; *p* = 0.0024) and in patients with diabetes (β = 32.88, CI, 15.21–50.55; *p* = 0.001).

**TABLE 6:** Uni- and Multi-variable regression to identify factors associated with the Stroop Colors Test (delay, s).

	Univariable			Multivariable (*n* = 60) Backward selection		
	Regression coefficient	95% C.I. (range)	*p*-Value	Regression coefficient	95% C.I. (range)	*p*-Value
Age	0.79	(0.21–1.36)	0.008	0.64	(0.09–1.19)	0.024
BMI (kg/m^2^)	0.08	(−0.67–0.83)	0.829			
Female gender	4.01	(−3.18–11.20)	0.269			
SAS	4.24	(−2.35–10.83)	0.203			
OSAS	1.27	(−5.33–7.87)	0.702			
CSAS	1.55	(−6.18–9.28)	0.689			
Apnea-hypopnea index (AHI)	0.24	(0.08–0.40)	0.003	0.17	(0.02–0.32)	0.030
**Sleep apnea severity**						
*None*	REF					
*Mild*	4.31	(−8.62–17.25)	0.507			
*Moderate*	3.21	(−6.17–12.60)	0.496			
*Severe*	8.38	(0.20–16.56)	0.045			
Blood oxygen saturation (SpO2,%)	−0.47	(−2.05–1.10)	0.552			
Sleep time with hypoxia^∗^ (%)	−0.03	(−0.26–0.21)	0.831			
Number of events per night	0.02	(−0.00–0.05)	0.063			
Diabetes	6.88	(−2.59–16.34)	0.151	7.91	(−0.47–16.29)	0.064
Inflammation	−1.53	(−8.20–5.14)	0.648			
Hypercholesterolemia^∗∗^	−5.46	(−18.68–7.75)	0.411			
Hypertriglyceridemia	−0.01	(−8.09–8.07)	0.998			
Vitamin D deficiency	−5.07	(−11.48–1.35)	0.119			
Anemia	6.93	(0.22–13.63)	0.043	5.98	(−0.07–12.04)	0.053

*^∗^Hypoxia defined as a reduction of SpO2 by 10%. ^∗∗^Excluded from multivariable analysis due to low incidence (<10).*

**TABLE 7:** Uni- and Multi-variable regression to identify factors associated with the Stroop Words Test (delay, s).

	Univariable			Multivariable (*n* = 56) Backward selection		
	Regression coefficient	95% C.I. (range)	*p*-Value	Regression coefficient	95% C.I. (range)	*p*-value
Age	1.06	(−0.13–2.25)	0.081			
BMI (kg/m^2^)	0.70	(−0.70–2.11)	0.321			
Female Gender	2.33	(−11.68–16.33)	0.741			
SAS	8.59	(−4.16–21.35)	0.182			
OSAS	7.23	(−5.36–19.82)	0.254			
CSAS	−2.35	(−17.34–12.64)	0.754			
Apnea-hypopnea index (AHI)	0.31	(0.00–0.62)	0.047	0.31	(0.00–0.62)	0.047
**Sleep apnea severity**						
*None*	REF					
*Mild*	15.59	(−8.58–39.76)	0.201			
*Moderate*		(−13.97–22.54)	0.640			
*Severe*	14.59	(−0.90–30.07)	0.064			
Blood oxygen saturation (SpO2,%)	−1.17	(−4.14–1.80)	0.433			
Sleep time with hypoxia^∗^ (%)	−0.19	(−0.64–0.26)	0.393			
Number of events per Night	0.04	(−0.01–0.09)	0.092			
Diabetes	3.51	(−16.95–23.97)	0.732			
Inflammation	−2.20	(−15.06–10.66)	0.733			
Hypercholesterolemia^∗∗^	9.81	(−14.91–34.53)	0.429			
Hypertriglyceridemia	0.32	(−15.34–15.99)	0.967			
Vitamin D deficiency	−12.08	(−24.33–0.16)	0.053			
Anemia	0.05	(−13.33–13.43)	0.994			

*^∗^Hypoxia defined as a reduction of SpO2 by 10%. ^∗∗^Excluded from multivariable analysis due to low incidence (<10).*

**TABLE 8:** Uni- and Multi-variable regression to identify factors associated with the Stroop Interference Test (delay, s).

	Univariable			Multivariable (*n* = 50) Backward selection		
	Regression coefficient	95% C.I. (range)	*p*-Value	Regression coefficient	95% C.I. (range)	*p*-value
Age	1.29	(−0.05–2.63)	0.059	1.13	(−0.05–2.30)	0.061
BMI (kg/m^2^)	0.12	(−1.41–1.66)	0.872			
Female Gender	5.74	(−9.76–21.24)	0.460			
SAS	16.93	(3.21–30.66)	0.017			
OSAS	1.98	(−12.05–16.01)	0.778	13.57	(−0.14–27.28)	0.052
CSAS	17.18	(2.02–32.33)	0.027	24.45	(8.42–40.49)	0.004
Apnea-hypopnea index (AHI)	0.45	(−0.11–0.78)	0.011			
**Sleep apnea severity**						
*None*	REF					
*Mild*	–6.02	(−26.26–14.23)	0.553			
*Moderate*	7.65	(−20.32–35.62)	0.585			
*Severe*	14.15	(−3.65–31.95)	0.116			
Blood Oxygen Saturation (SpO2,%)	–0.33	(−3.58–2.93)	0.840			
Sleep Time with hypoxia^∗^ (%)	0.03	(−0.44–0.51)	0.890			
Number of events per Night	0.04	(−0.02–0.09)	0.189			
Diabetes	28.53	(−8.65–48.41)	0.006	32.88	(15.21–50.55)	0.001
Inflammation	–0.52	(−14.80–13.77)	0.942			
Hypercholesterolemia^∗∗^	–12.64	(−42.31–17.02)	0.395			
Hypertriglyceridemia	13.97	(−2.22–30.16)	0.089			
Vitamin D deficiency	–6.85	(−20.71–7.02)	0.326			
Anemia	8.65	(−5.91–23.21)	0.238			

*^∗^Hypoxia defined as a reduction of SpO2 by 10%. ^∗∗^Excluded from multivariable analysis due to low incidence (<10).*

## Discussion

The prevalence of SAS in elderly patients admitted to our geriatric unit was 63% (OSAS in 41% and CSAS in 22%), which is considerably higher than that reported in the general population, between 24 and 40% (Peppard et al., 2013; Heinzer et al., 2015). Our results demonstrated the preeminence of SAS among factors associated with cognitive deficit thus confirming previous observations from the literature (Olaithe and Bucks, 2013; Aoki et al., 2014; Arli et al., 2015; Osorio et al., 2015). It is worth noting that the prevalence of SAS in our cohort remains higher than recent estimates for the elderly population (25% for men and 42% for women) (Heinzer et al., 2015), which could be due to our great proportion of patients with cognitive deficit (Moran et al., 2005; Emamian et al., 2016). Interestingly, this finding is in agreement with recent observations from German geriatric wards (Gronewold et al., 2019). This study is nevertheless the first to investigate comorbidities associating with SAS while differentiating OSAS and CSAS in this particular population.

The patients in this cohort presented various levels of cognitive deficit, although all were diagnosed with disorders ranging from MCI to AD or vascular dementia. Interestingly, in this diverse cohort, OSAS was identified as an independent predictor of poor MDRS scores but was not associated with MMSE scores. While both tests are used to detect cognitive impairment in the elderly, there are indications that they may not evaluate the same cognitive domains (Freidl et al., 1996), or that the MDRS may be more specific and sensitive (Kaszas et al., 2012; Holden et al., 2016). Furthermore, the ability of the MMSE to efficiently screen for cognitive impairment in the elderly has recently been put into question (Gagnon et al., 2018). It is important to note that the fact that CSAS did not affect MDRS scores in our study could be due to its lower incidence in our cohort and is no proof of lack of effect. Our results also revealed that AHI associates with impaired attention, indicated by the increased delay in the Stroop Colors and Words tests, and that CSAS and perhaps OSAS associate with impaired executive function, indicated by the increased delay in the Stroop Interference test. This is in line with the literature reporting that SAS affects vigilance and the attentional domain (Beebe et al., 2003) and executive function (Beebe et al., 2003; Olaithe and Bucks, 2013). In 2012, Bucks et al. (2013) suggested that OSAS may affect vigilance through sleep fragmentation and executive function through hypoxia. In this study, we measured hypoxia and sleep apnea severity (AHI) separately but were not able to verify this hypothesis. It is unclear why, unlike previous literature, AHI rather than oxygen desaturation was associated with cognitive deficit in our cohort, though it could be due to the different manifestations in SAS in the elderly population and in the general population (Al Lawati et al., 2009; Heinzer et al., 2015). The relationship between intermittent hypoxia and cognition remain complex, however, and animal studies have suggested that intermittent hypoxia may not directly impair brain structure (Jorba et al., 2017), but may play a role in the etiology of Alzheimer’s Disease (Shiota et al., 2013; Menal et al., 2018; Macheda et al., 2019). Nevertheless, fragmented sleep and excessive daytime sleepiness have been shown to modify the relationship between AHI and several co-morbidities (Nena et al., 2012; Ren et al., 2016) and could contribute to cognitive decline in SAS patients (Zhou et al., 2016). Unfortunately, daytime sleepiness was not evaluated in this study, and could therefore not be included in the multivariable model. We found no association between the number of events per night and cognitive outcomes, though this variable may have underestimated sleep fragmentation, as it did not take snoring into account. The interaction between AHI, sleep fragmentation, daytime sleepiness, and cognitive decline in the elderly remains therefore a question for further search.

We decided to use vascular risk factors known to associate with SAS or AD to adjust the regression models for our cognitive tests. Interestingly, only diabetes independently associated with reduced MMSE and MDRS scores, as well as increased Stroop delay. Taken together, OSAS and diabetes are associated with a reduction of 26 points in the MDRS. Interestingly, severe sleep apnea was shown to be associated with diabetes in the general population (Heinzer et al., 2015). This preeminence of diabetes among risk factors for cognitive deficit has been recently demonstrated (Musen et al., 2018) and our results indicate that sleep apnea should be investigated among the other risk factors for the cognitive deficit of the elderly. Indeed, sleep apnea treatment with continuous positive air pressure has shown success in slowing down cognitive decline in the elderly (Kushida et al., 2012; Troussiere et al., 2014; Osorio et al., 2015), so that sleep apnea could be considered a modifiable risk factor for cognitive decline in the elderly (Ancoli-Israel et al., 2008).

This study is among the first to combine a comprehensive measure of sleep apnea, including the AHI, sleep hypoxia and events per night, with a multivariable regression analysis including known vascular risk factors to determine associations with cognitive deficit in cohort of elderly patients suffering from diffuse cognitive impairment. Moreover, the cohort was controlled for depression and anxiety. The fact that sleep apnea was selected among other well-established risk factors is a powerful demonstration of its association with cognitive deficit in these patients and suggests that sleep apnea ought to be screened for and treated in priority when possible. Further, our distinction of patients suffering primarily from OSAS or CSAS offers novel insight into the effects of these different, yet oftentimes overlapping disorders.

However, this study has several limitations. First, the age of the patients and their cognitive impairment caused a large number of failures of the neurocognitive assessment, especially for the Stroop test. This has weakened our regression analyses and increased the chance of underestimating associations. Second, the fact that patients unable to complete the neurocognitive tests were excluded from the study has created a potential bias, since these patients were likely to exhibit more severe cognitive symptoms. Third, no additional measure of dementia was used to validate MDRS results and assess dementia severity. Fourth, the prevalence of women in our cohort represents the population admitted to our institution, and our results may not be applicable for younger sleep apnea patients, which are predominantly men. Finally, our list of competing risk factors was incomplete, as we were unable to correctly or systematically assess the patients’ smoking habits, alcohol consumption, physical inactivity, or daytime sleepiness.

## Conclusion

This study reveals the high prevalence of sleep apnea in elderly patients with cognitive impairment, and demonstrates the independent association between sleep apnea and cognitive deficit. OSAS was associated with a reduction of the MDRS score, while CSAS was associated with an increased Stroop Interference delay. Our data suggest that elderly patients with cognitive deficit should be screened for sleep apnea and encouraged to follow treatments, if necessary, to prevent cognitive decline.

## Data Availability Statement

The datasets generated for this study are available on request to the corresponding author.

## Ethics Statement

This work was completed after being approved by an independent Ethical Review Board (CPP Amiens: 2015/6) and the national Data Protection Authority (CNIL:150075B-31). The study was registered in clinicaltrials.gov (NCT02578303) and has not been submitted to any other journal.

## Author Contributions

JA-Z designed the study, collected the data, and edited the manuscript. J-MS designed the study and edited the manuscript. JV contributed to statistical analyses, interpretation of data, and manuscript writing. MS interpreted the data, wrote and edited the manuscript. YD collected the data and edited the manuscript. KM contributed to statistical analyses and manuscript editing. OB designed the study, interpreted the data, and wrote and edited the manuscript. All authors approved and accountability for final version of the manuscript.

## Conflict of Interest

MS and JV were employed by ReSurg SA. The remaining authors declare that the research was conducted in the absence of any commercial or financial relationships that could be construed as a potential conflict of interest.
